# Arginine improves peroxisome functioning in cells from patients with a mild peroxisome biogenesis disorder

**DOI:** 10.1186/1750-1172-8-138

**Published:** 2013-09-09

**Authors:** Kevin Berendse, Merel S Ebberink, Lodewijk IJlst, Bwee Tien Poll-The, Ronald J A Wanders, Hans R Waterham

**Affiliations:** 1 Department of Clinical Chemistry, Laboratory Genetic Metabolic Diseases, Academic Medical Center, University Hospital of Amsterdam, Meibergdreef 9, 1105 AZ Amsterdam, The Netherlands; 2 Department of Pediatric Neurology, Emma Children’s Hospital, Academic Medical Center, University Hospital of Amsterdam, Meibergdreef 9, 1105 AZ Amsterdam, The Netherlands

**Keywords:** Peroxisome biogenesis disorder, Zellweger spectrum disorder, Misfolded protein, Peroxisomal mosaicism, Arginine, Therapy

## Abstract

**Background:**

Zellweger spectrum disorders (ZSDs) are multisystem genetic disorders caused by a lack of functional peroxisomes, due to mutations in one of the *PEX* genes, encoding proteins involved in peroxisome biogenesis. The phenotypic spectrum of ZSDs ranges from an early lethal form to much milder presentations. In cultured skin fibroblasts from mildly affected patients, peroxisome biogenesis can be partially impaired which results in a mosaic catalase immunofluorescence pattern. This peroxisomal mosaicism has been described for specific missense mutations in various *PEX* genes. In cell lines displaying peroxisomal mosaicism, peroxisome biogenesis can be improved when these are cultured at 30°C. This suggests that these missense mutations affect the folding and/or stability of the encoded protein. We have studied if the function of mutant PEX1, PEX6 and PEX12 can be improved by promoting protein folding using the chemical chaperone arginine.

**Methods:**

Fibroblasts from three *PEX1* patients, one *PEX6* and one *PEX12* patient were cultured in the presence of different concentrations of arginine. To determine the effect on peroxisome biogenesis we studied the following parameters: number of peroxisome-positive cells, levels of PEX1 protein and processed thiolase, and the capacity to β-oxidize very long chain fatty acids and pristanic acid.

**Results:**

Peroxisome biogenesis and function in fibroblasts with mild missense mutations in *PEX1, 6* and *12* can be improved by arginine.

**Conclusion:**

Arginine may be an interesting compound to promote peroxisome function in patients with a mild peroxisome biogenesis disorder.

## Background

Peroxisomes are ubiquitous organelles bound by a single membrane and present in nearly all eukaryotic cells. There are approximately 50 different peroxisomal enzyme proteins, which are involved in various biochemical pathways and can be present in different types of cells. Among others, peroxisomes are involved in the degradation of Very Long Chain Fatty Acids (VLCFA; ≥C_22_:_0_), the formation of bile acids, the synthesis of plasmalogens and the oxidation of phytanic acid [1]. Peroxisome biogenesis disorders (PBDs) are autosomal recessive disorders characterized by an impairment in one or more peroxisomal functions. The PBDs can be divided into two subtypes; the Zellweger Spectrum Disorders (ZSD, OMIM #601539) and rhizomelic chondrodysplasia punctata type I (RCDP, OMIM #215100). Clinically and biochemically, the ZSD represent a continuum of at least three phenotypes, including Zellweger syndrome (ZS, OMIM #214100) as the most severe, neonatal adrenoleukodystrophy (NALD, OMIM #202370), and infantile Refsum disease (IRD, OMIM # 266510) [2-4] as the least severe phenotype.

In ZS, there is a complete loss of peroxisomal functions. In contrast, cells from NALD and IRD patients still contain functional peroxisomes albeit reduced in number. When skin fibroblasts from milder patients are cultured at 37°C and then examined for the localization of the peroxisomal matrix protein catalase, a mixed population of cells with either catalase-containing or catalase-lacking peroxisomes can be seen [5-7]. This phenomenon is called peroxisomal mosaicism and has been described for mutations in various *PEX* genes (e.g. *PEX1, PEX2, PEX6, PEX10* and *PEX12*), which are associated with a relatively mild phenotype [5,6,8,9].

Proteins encoded by *PEX* genes are called peroxins and play a role in normal peroxisome assembly. Currently 14 peroxins are known to be involved in this process and mutations in 13 of the *PEX* genes have been associated with ZSDs [9,10]. When skin fibroblasts, displaying peroxisomal mosaicism, are cultured at 30°C, all cells regain catalase-containing peroxisomes. In contrast, when the fibroblasts are cultured at 40°C, all cells lose catalase-containing peroxisomes [5] which resembles the peroxisomal phenotype in fibroblasts from classical Zellweger patients. This suggests that the mutations associated with peroxisomal mosaicism cause an unstable and/or incorrectly folded PEX protein. Moreover, these observations also suggest that improving the folding of the mutated protein, e.g. by lowering the temperature, can result in the restoration of peroxisome biogenesis. Here, we have studied whether the chemical chaperone arginine is also capable of improving peroxisome biogenesis in fibroblasts displaying peroxisomal mosaicism.

Chemical chaperones are small-molecule osmolytes, which have the capacity to improve protein folding. Previous studies have shown that several compounds are capable of improving peroxisomal function [11]. Arginine has been described to correct protein folding and suppress protein aggregation *in vitro*[12]. Furthermore, it has been shown that arginine supplementation restored PDHc function in a patient with pyruvate dehydrogenase deficiency [13]. The mechanism which underlies its chaperone function is unknown, but might involve conformational correction and prevention of nonproductive protein interactions [14].

In this study, we show that arginine is able to improve peroxisome biogenesis and functioning in cells from patients with a mild ZSD, that display peroxisomal mosaicism due to mild mutations in the *PEX1, PEX6* or *PEX12* gene.

## Material and methods

### Cell culture

For this study we used primary skin fibroblasts from different patients with a mild peroxisomal disease. Three cell lines were homozygous for the c.2528G>A (p.G843D) mutation in the *PEX1* gene (PEX1-G843D); one cell line was compound heterozygous for the c.821C>T (p.P274L) and c.1314_1321delGGAGGCCT (p.E439fsX3) mutation in *PEX6* and one cell line was homozygous for the c.959C>T (p.S320F) mutation in the *PEX12* gene. As negative control we used a cell line homozygous for the c.2097insT (p.I700fsX41) mutation in *PEX1* (PEX1-I700fsX41), which has no functional peroxisomes (Zellweger syndrome). In accordance with the institutional guidelines and the Dutch Code of Conduct, identifiable clinical and personal data from the patients were not available for this study. The cell lines were cultured in 10% Dulbecco’s Modified Eagle’s Medium (DMEM) supplemented with 10% fetal bovine serum, 25 mM HEPES buffer, 100 U/ml penicillin, 100 μg/ml streptomycin and amphotericin 250 μg/ml. All cultures were maintained at 37°C in a humidified atmospheric environment with 5% CO_2_ in T162, T75 culture flasks or in six-well plates (for immunofluorescence). Cells were harvested by use of trypsin (0.5% trypsin-EDTA, Invitrogen), washed once with phosphate-buffered saline (PBS) (Fresensius Kabi Nederland B.V.) and twice with 9 g/L NaCl (Fresensius Kabi Nederland B.V). Passage numbers of the PEX1_1, PEX1_2, PEX1_3, ZS, PEX6, PEX12 and control cell lines were 16–19, 16–20, 13–16, 15–20, 7–8, 19–20 and 10–20, respectively.

### Cell incubations

Fibroblasts were incubated for different time periods as indicated, with 5, 10 or 20 mM of L-arginine monohydrochloride (MERCK, Darmstadt, Germany), 20 mM L-glutamine (MERCK, Darmstadt, Germany) or 543 mM (=50 g/L) glycerol (ACROS Organics, Geel, Belgium) added to the culture medium. Arginine, glutamine and glycerol were dissolved directly in the culture medium and sterilized through a 0.45 μm filter (Millipore Millex-HP). Endogenous levels of L-arginine and L-glutamine in the medium was 3 mM and 4.4 mM respectively. Every seven days, the cells were subcultured at a 1:2 dilution and fresh medium was added.

### Catalase immunofluorescence (IF) microscopy

Catalase IF was performed essentially as described previously [15]. Fibroblasts were plated in six-well plates containing ethanol-sterilized cover slips. The next day (when the fibroblasts were attached), the cells were incubated with the different supplements as indicated and prepared for catalase immunofluorescence (IF) microscopy. To determine the effectiveness of the supplementation, the percentage of peroxisome-positive cells (cells with punctate catalase fluorescence) was determined by analyzing at least 200 cells in duplicate. In each experiment the microscope slides were coded to prevent bias.

### Immunoblot analysis

Fibroblast pellets used for protein analysis, were dissolved in a volume of 200 μl of lysis buffer (PBS, 0.25% Triton X-100, protease inhibitor cocktail tablet (Roche, Mannheim, Germany lot# 13690100)). Protein samples were sonicated twice (8 watt, 40 Joule) on ice water. Protein concentration was determined by the BCA protein assay according to the manufacturer’s (Pierce, Rockford, USA) protocol using human serum albumin (HSA) as standard.

Immunoblot analysis of peroxisomal thiolase was done according to a published method [16].

Immunoblot analysis was performed with homogenates of cultured fibroblasts (50 μg protein), separated by 10% SDS-PAGE and transferred onto nitrocellulose by semidry blotting. Antiserum against ACAA1 (thiolase) (Sigma-Aldrich, St Louis, Missouri, USA) and PEX1 (BD Transduction laboratories, Franklin Lakes, New Jersey, USA) were used at a 1:2000 and 1:250 dilution respectively. As a control for equal protein loading, we simultaneously probed the PEX1 immunoblot with a monoclonal antibody against α-tubulin (Sigma-Aldrich, St Louis, Missouri, USA), using a 1:10000 dilution. Antigen-antibody complexes were visualized with IRDye 800CW goat anti-rabbit secondary antibody for ACAA1, IRDye 800CW goat anti-mouse antibody for PEX1, and IRDye 680RD donkey anti-mouse secondary antibody for tubulin using the Odyssey Infrared Imaging System (LI-COR Biosciences, Nebraska, USA). Quantification of thiolase and PEX1 immunoblots was done using AIDA Image analyzer software (Version 4.26, Raytest, Straubenhardt, Germany), which allows sensitive and reliable quantification of protein amounts in a non-saturated manner.

### β-oxidation of D_3_-C22:0 and pristanic acid

Degradation (to D_3_-C16:0) and elongation (to D_3_-C26:0) of D_3_-VLCFA in intact cells was measured using D_3_-C22:0 as substrate [17]. D3-C22:0 (i.e deuterium-labeled free 22,22,22-D3-docosanoic acid) was purchased from CDN Isotopes (Pointe-Claire Canada, lot #R259AP3).

Assays were performed in duplicate in T75 culture flasks. Medium was replaced by fresh medium supplemented with D_3_-C22:0 (6 mM dissolved in DMSO) at a final concentration of 30 μM. After 72 hours, cells were harvested and VLCFA analysis was done as described before [18]. For determination of protein concentrations, cells were resuspended in 200 μl deionized water and sonicated for 10 seconds (7 Watt). Pristanic acid β-oxidation was measured radio chemically according to a published method [19].

## Results

We studied the effect of arginine on peroxisome biogenesis and functioning in primary fibroblasts carrying mild mutations in *PEX1*, *PEX6* or *PEX12*. As a positive control we used glycerol, which we and others previously found to have a positive effect on these parameters (not shown) [11]. In all cell lines the mutations were found to result in peroxisomal mosaicism when the cells were cultured at 37°C. As a negative control we used fibroblasts from a ZS patient (homozygous *PEX1*-I700fsX41), which are completely peroxisome-deficient and as positive control we used three cell lines from unaffected individuals. Initially, we focused on cells with *PEX1* mutations, because the *PEX1* gene is by far the most commonly affected gene in ZSDs, with the homozygous p.G843D mutation accounting for one-tenth of all *PEX1* mutations [8].

### Arginine restores peroxisome biogenesis in *PEX1*-G843D fibroblasts

**Figure 1:**
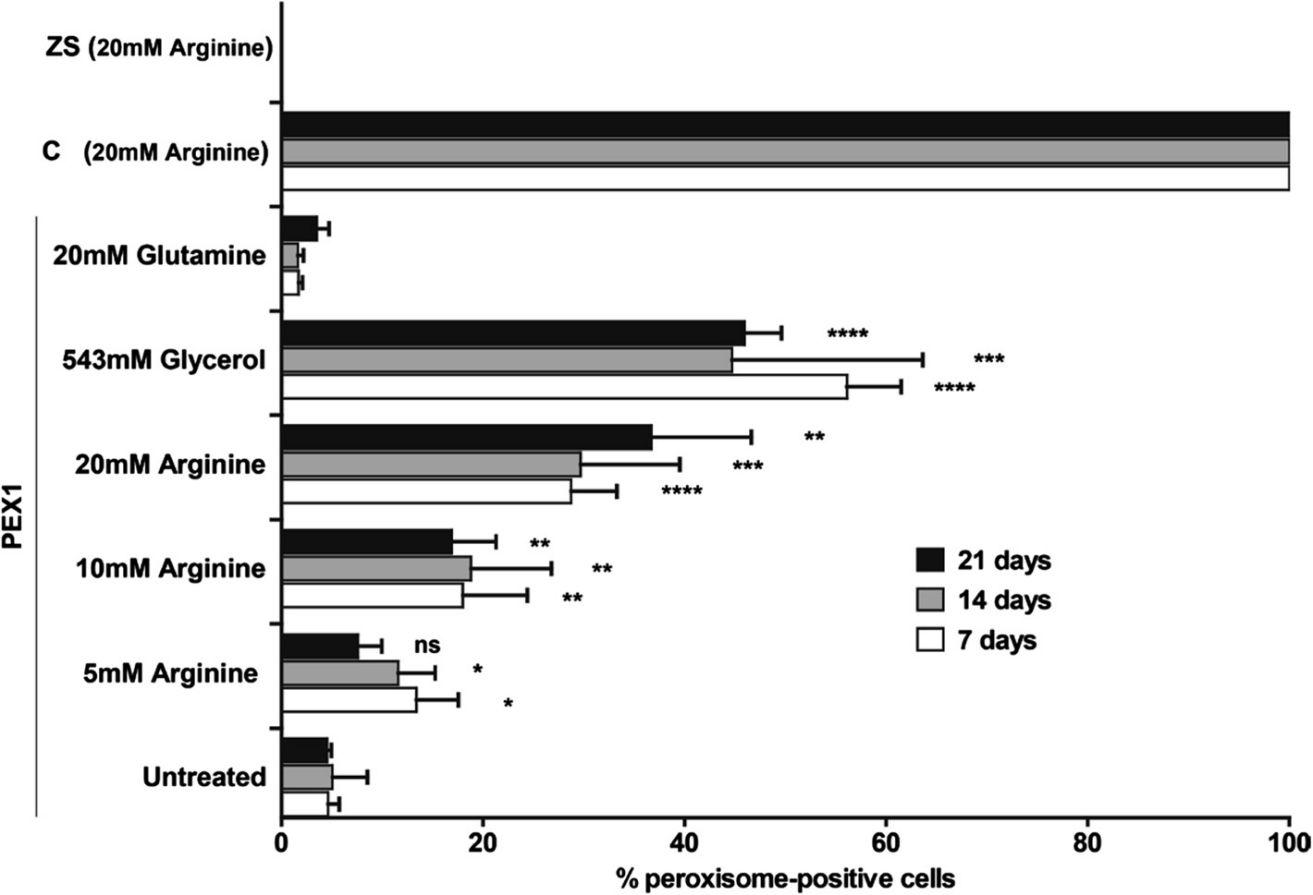
**Catalase immunofluorescence of fibroblasts incubated with arginine.** PEX1-G843D patient fibroblasts were incubated for 7, 14 or 21 days with different concentrations of arginine, glycerol or glutamine. Glycerol was used as a positive control. In four independent experiments, two cover slips per condition were examined for the number of cells with catalase-containing peroxisomes among at least 200 cells. Data are presented as means±SD, statistical analysis was performed with a two-tailed Student’s *t*-test, *, P < 0.05; **, P < 0.005; ***, P < 0.0005; ****, P < 0.0001 versus untreated PEX1-G843D fibroblasts. Two additional PEX1-G843D cell lines showed similar results (Additional file 1: Figure S1 and Figure 4). Control (C) showed 100% peroxisome-positive cells. PEX1-I700fsX41 (negative control = ZS) displayed no peroxisome-positive cells.

We first studied the effect of 50 g/L glycerol (positive control [11]) and 5, 10 and 20 mM arginine on the number of cells with catalase-containing peroxisomes (i.e. peroxisome-positive cells) among a total of at least 200 cells (Figure 1). We observed significant increases in the number of peroxisome-positive cells among fibroblasts supplemented with arginine compared to untreated fibroblasts (Figure 1 and Additional file 1: Figure S1). Growth of fibroblasts in the presence of different concentrations of arginine resulted in an increase in the number of peroxisome-positive cells in a concentration-dependent manner.

To exclude a positive effect as consequence of osmotic stress, we also incubated the cells with glutamine, which did not have any effect on the number of peroxisome-positive cells. Supplementation of arginine to the PEX1- I700fsX41 cells did not result in peroxisome-positive cells either, whereas in control fibroblasts we observed 100% peroxisome-positive cells. Because the increase in the number of peroxisome-positive cells when supplemented with 5 mM arginine, was not significant, we omitted this concentration in our further experiments.

**Figure 2:**
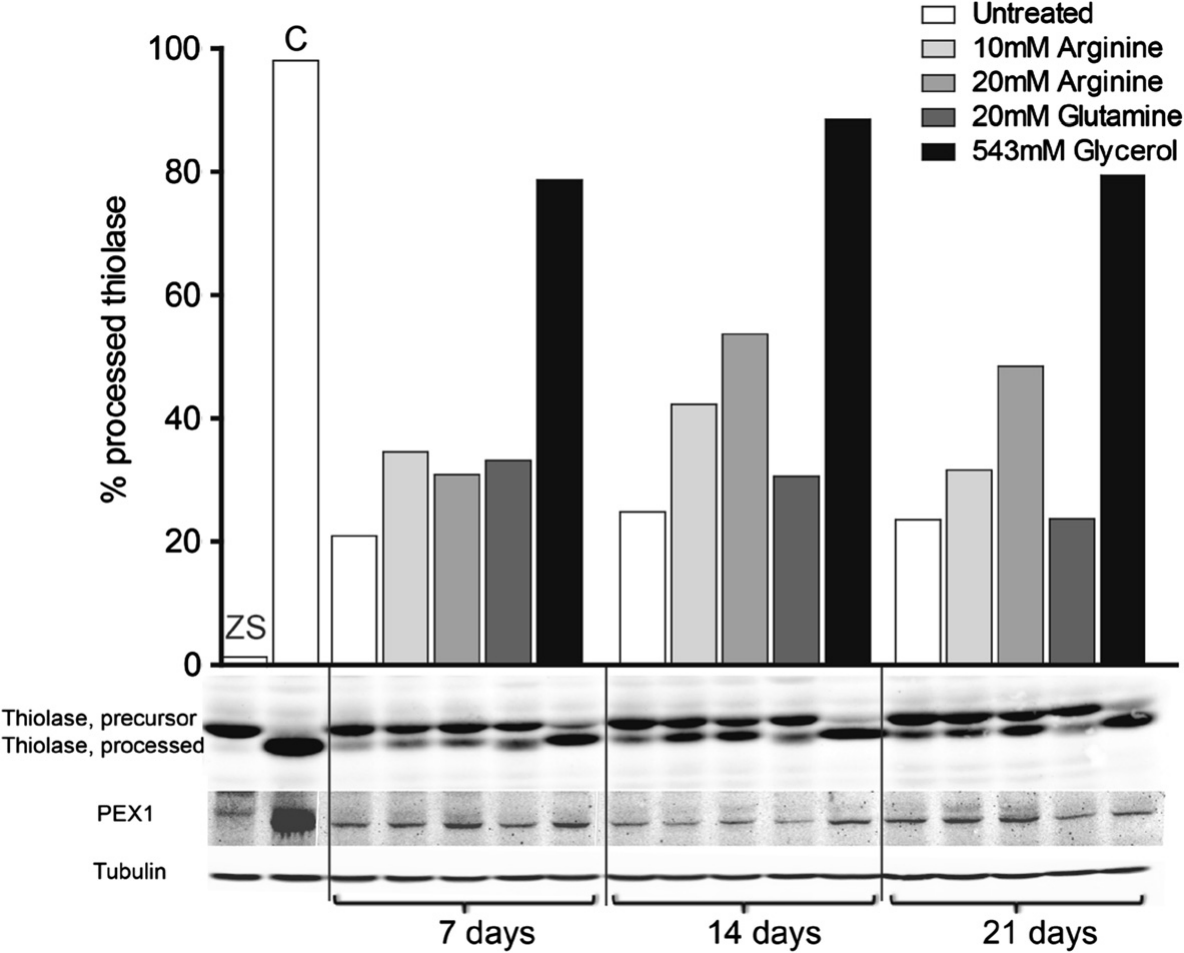
**Thiolase and PEX1 immunoblots.** The effect of 7, 14 and 21 days incubation of PEX1-G843D cells with arginine, glycerol and glutamine, on the amount of processed (41 kDa) peroxisomal thiolase and PEX1 protein (143 kDa) was studied by immunoblotting using cell homogenates. The precursor of thiolase, 44 kDa, is processed in peroxisomes to a mature peptide of 41 kDa. Control showed 98% processed thiolase, the severe cell line showed 1% processed thiolase. The faint upper band, seen in the ZS cell line with PEX1 antibody, is aspecific. Tubulin was used to confirm equal protein loading. Data shown are representative of two independent experiments and two additional PEX1-G843D cell lines (data not shown). ZS = severe PEX1-I700fsX41, C = Control.

In addition to the number of peroxisome-positive cells accessed by catalase fluorescence, we also determined the extent of intraperoxisomal processing of the peroxisomal protein thiolase by immunoblots analysis (Figure 2). The precursor of thiolase, 44 kDa, is proteolytically processed in the peroxisome to a mature peptide of 41 kDa, as mediated by TYNSD1 [20]. After 7 days of incubation with arginine no increase in the amount of processed 41 kDa thiolase was observed when compared to untreated cells (Figure 2). However, after 14 and 21 days of incubation with arginine or glycerol, an increasing amount of thiolase was found to be processed in the *PEX1*-G843D cell lines, indicating improved peroxisomal import and protein processing. The addition of arginine resulted in increased levels of processed thiolase (41 kDa) in a concentration and time-dependent manner, while glutamine supplementation showed no effect (Figure 2). The *PEX1*-I700fsX41 fibroblasts only showed unprocessed precursor thiolase of 44 kDa under all conditions (data not shown).

Subsequent to thiolase immunoblot analysis we examined the total levels of PEX1 protein by immunoblot analysis, to evaluate if arginine and glycerol supplementation had an effect on the amount of PEX1 (Figure 2). Compared to untreated cells, we observed a minor increase in PEX1 levels in mild *PEX1* fibroblasts incubated with 20 mM arginine for 21 days. In cells supplemented with glycerol we already noted an effect after 14 days of incubation. Because the increase in the amount of processed thiolase in cells incubated for 7 days was not significant, we omitted this time point in our further experiments.

### Arginine ameliorates the metabolic functions of peroxisomes in *PEX1*-G843D fibroblasts

Patients with a mild ZSD have a (partial) deficiency in the degradation of VLCFA (≥C22:0) due to a defect in the peroxisomal β-oxidation, in contrast to ZS patients in which functional capacity is fully abolished [21]. Therefore, we investigated whether the peroxisomal VLCFA β-oxidation, i.e. β-oxidation of D_3_-C22:0 to D_3_-C16:0 [17], in the *PEX1*-G843D cell lines can be restored by addition of arginine and glycerol. It is also known that, due to the defective peroxisomal β-oxidation, patients with a PBD elongate the accumulating VLCFA (to C26:0) [22]. Hence, we also studied the elongation of D_3_-C22:0 to D_3_-C26:0.

**Figure 3:**
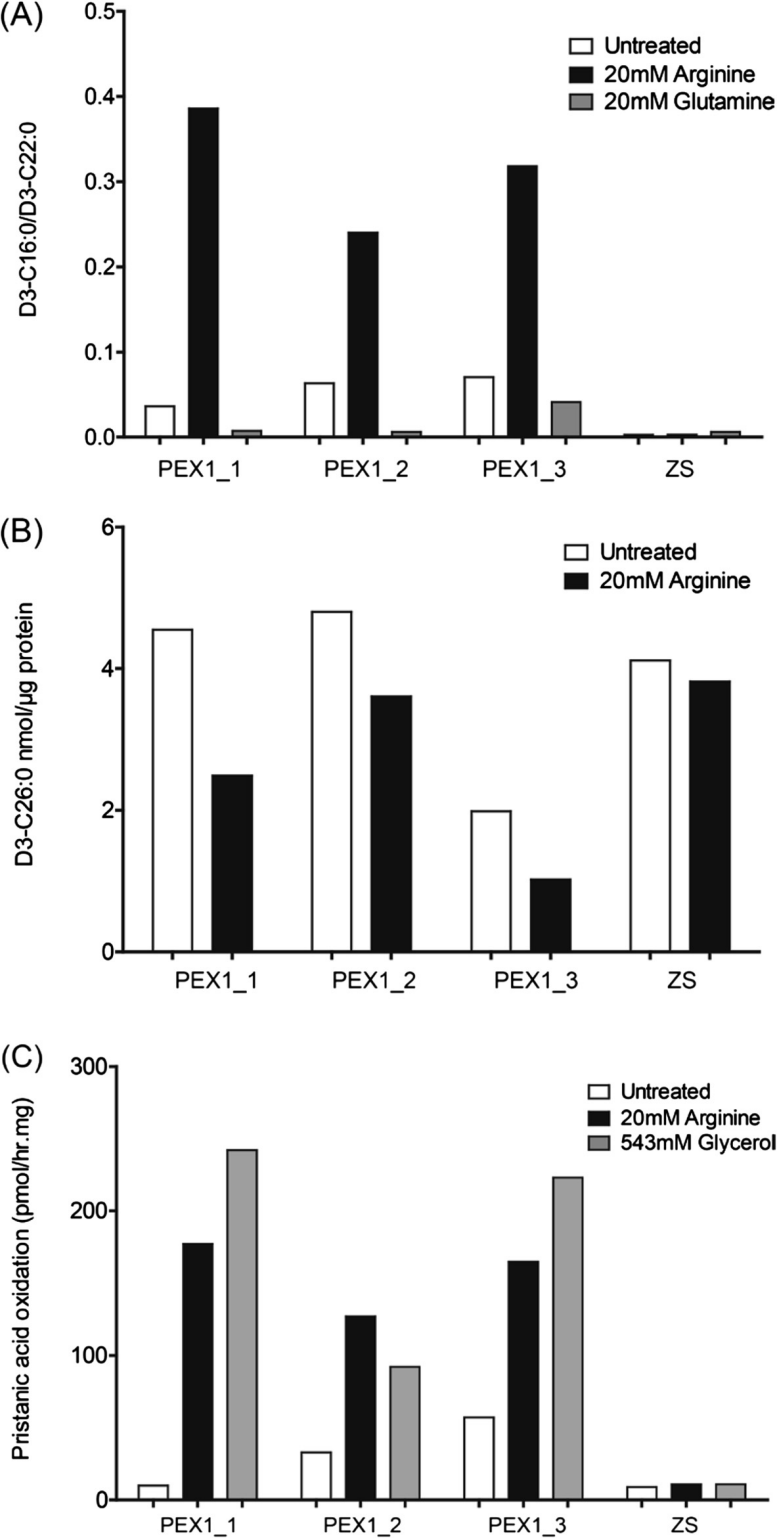
**Effect of arginine supplementation on peroxisomal D3-C22:0, pristanic acid β-oxidation and D3-C22:0 elongation. (A)** The D3-C16:0/D3-C22:0 ratio (degradation) and **(B)** total amount of D3-C26:0 (elongation) was determined in three independent PEX1-G843D cell lines, incubated for 21 days with arginine. The D3-C16:0/D3-C22:0 ratio and total amount D3-C26:0 in control cells was 2.5 (±0.5) and 0.2 (±0.1) nmol/μg protein, respectively. In fibroblasts from the severe PEX1-I700fsX41 (ZS), the ratio and total amount was 0.002 (±0.001) and 4.2 (±0.1) nmol/μg protein, respectively. **(C)** The effect of 14 days arginine incubation on the pristanic acid β-oxidation in three PEX1-G843D cell lines (PEX1_1, PEX1_2 and PEX1_3). The pristanic acid β-oxidation in control cells was 544 (±182) pmol/hr.mg. Statistical analysis was performed with a two-tailed Student’s *t*-test, p-values, when grouped, <0.0001 (figure **A**), 0.007 (figure **B**) and 0.006 (figure **C**). Trend in improvement of D3-C22:0 degradation and pristanic acid β-oxidation is representative for a second independent experiment.

Figure 3A shows the ratio of D_3_-C16:0/D_3_-C22:0 (used as an indication for peroxisomal β-oxidation capacity) in three independent *PEX1*-G843D cell lines. Incubation with 20 mM arginine resulted in a 4.5 to 10 fold increase in the β-oxidation of D_3_-C22:0 in the three PEX1 cell lines, indicating improved peroxisomal functioning. No effect of arginine supplementation on peroxisomal β-oxidation in control and ZS fibroblasts was observed. Furthermore, incubation with glutamine did not show an effect on the β-oxidation of D_3_-C22:0 to D_3_-C16:0. In addition to an increased peroxisomal β-oxidation, the arginine supplementation also leads to decreased elongation rates of D_3_-C22:0 to D_3_-C26:0 (Figure 3B).

Because ZSD patients also have a (partial) defect in the β-oxidation of pristanic acid [23,24], we also studied the effect of arginine and glycerol on peroxisomal pristanic acid oxidation. *PEX1*-G843D cell lines showed a residual pristanic acid β-oxidation capacity of approximately 10 to 50 pmol/hr.mg. However, upon arginine incubation, the fibroblasts showed a 3 to 15 fold increase in pristanic acid β-oxidation capacity (Figure 3C). Glycerol caused an even larger increase to approximately 50 to 220 pmol/hr.mg. Fibroblasts from the ZS cell line showed no increase in pristanic acid β-oxidation when incubated with arginine or glycerol.

### Improvement of peroxisomal biogenesis in *PEX6* and *PEX12* mutant fibroblasts

**Figure 4:**
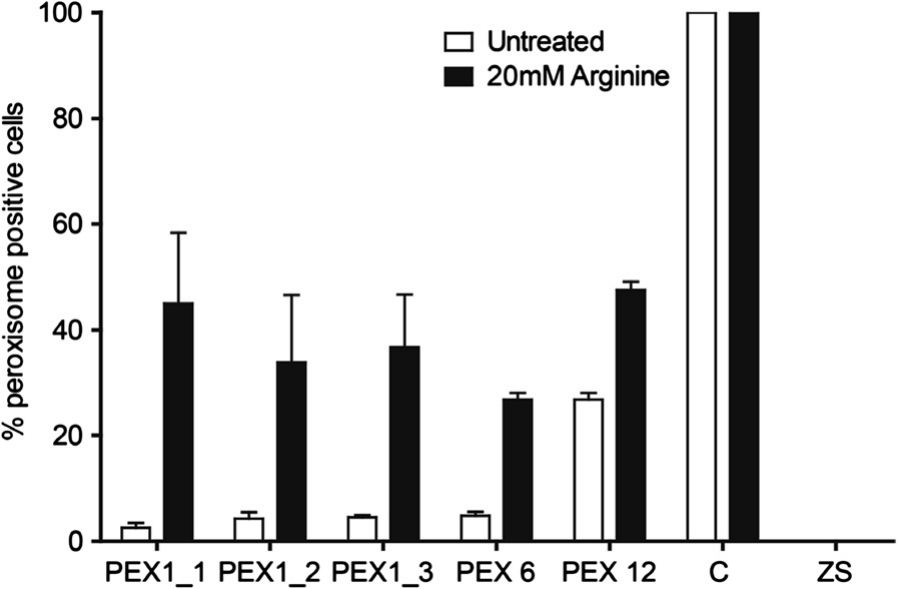
**Catalase immunofluorescence in PEX1, PEX6 and PEX12 deficient fibroblasts incubated with arginine.** Shown is the effect of 21 days incubation with 20 mM arginine on the three different PEX1-G843D cell lines and a PEX6 and a PEX12 patient cell line. Two independent cover slips per condition were examined by counting the number of cells with catalase-containing peroxisomes among at least 200 cells. Control (C) showed 100% peroxisome-positive cells. PEX1-I700fsX41 (negative control = ZS) displayed no peroxisome-positive cells. The data are presented as mean±SD.

Following the positive effects in the *PEX1* cell lines, we also investigated the effect of arginine in specific *PEX6* and *PEX12* cell lines. Both cell lines display peroxisomal mosaicism, with *PEX12*-p.S320F being the most common *PEX12* mutation. This mutation is common in patients from Turkish descent [8]. As observed in the *PEX1* cell lines, the addition of 20 mM arginine for 21 days resulted in an increase in the number of peroxisome positive cells (Figure 4) indicating that the effect of arginine is not mutation specific.

## Discussion

Currently, there is no cure for ZSDs and possibilities for supportive and symptomatic treatment are limited. Some patients received docosahexaenoic acid without any proven positive effect [25]. In addition, some patients follow a phytanic acid reduced diet and are supplemented with fat-soluble vitamins [26]. Previous studies revealed a close correlation between the clinical/biochemical severity and the functional consequences of the *PEX* mutations on the encoded protein in cells from patients with a PBD [27]. Among the reported mutations in the *PEX1* gene, which is most commonly defective, the *PEX1*-G834D mutation is associated with a mild clinical phenotype and relatively mild biochemical and cellular abnormalities [27-30].

In the present study, we investigated the effect of arginine and glycerol on peroxisome biogenesis and functioning in a subset of specific *PEX1*, *PEX6* or *PEX12-*defective fibroblasts, that all display peroxisomal mosaicism. Previous studies have shown that these cell lines showed improved peroxisomal function, when cultured at 30°C, suggesting a problem in protein folding. Furthermore Zhang et al., 2010 [11] observed a positive effect of several compounds on peroxisomal function in cell lines with the *PEX1*-G834D mutation. To study whether the presumed protein folding problem can be improved, we incubated these cell lines with arginine or glycerol, two known chemical chaperones *in vitro*[12].

Our results show a significant increase in the amount of peroxisome-positive cells, the intraperoxisomal processing of thiolase, the β-oxidation of D_3_-C22:0 (to D_3_-C16:0) and pristanic acid and a decrease in the elongation of D_3_-C22:0 (to D_3_-C26:0) in homozygous *PEX1*-G843D fibroblasts incubated with arginine. The mild untreated *PEX1* cell lines showed similar values of D_3_-C26:0 compared to the ZS cell line. This implies that untreated mild *PEX1* cell lines are not sufficiently metabolic active to rescue the elongation of D_3_-C22:0 to D_3_-C26:0. However, upon arginine supplementation, we observed decreased levels of D_3_-C26:0 levels. Furthermore, we found a positive effect of arginine on the amount of peroxisome-positive cells in specific *PEX6* and *PEX12* cell lines.

Despite the apparent improvement in peroxisome biogenesis and functioning, we only found a small increase in the amount of PEX1 protein upon immunoblot analysis of cell homogenates incubated for 21 days with 20 mM arginine. Apparently, the improvement in peroxisomal function is not accompanied by an obvious increase in total amount of PEX1 protein. Unfortunately, with immunoblot analysis, we cannot discriminate between incorrectly and correctly folded PEX1 protein, but we assume that arginine results in higher levels of correctly folded PEX1-G843D protein.

Osmotic stress can induce heat shock proteins, which enhance improved protein folding [31]. To exclude that the improvement of arginine was due to such an osmotic stress effect, we incubated the cells with comparable concentrations of glutamine. Glutamine is also an amino acid, but has no known chaperone properties. Overall, we did not observe any effect on peroxisome biogenesis and functioning upon glutamine supplementation.

## Conclusion

Although incubation with glycerol showed a more potent effect than arginine, the concentration used to reach this effect is too high (i.e. 543 mM) for potential clinical application. However, because plasma arginine concentrations can reach levels from up to 6 mM in humans and arginine supplementation is given to other diseases, including MELAS-syndrome [32], for which few side-effect are reported, we believe that arginine supplementation may provide a potential therapy for mild ZSD patients. In MELAS syndrome, arginine is thought to function as a nitric oxide stimulator rather than a chemical chaperone. It has been suggested that arginine activates the production of nitric oxide and thereby the induction of stress and upregulation of heat shock proteins [33]. It is possible that also in our study arginine activates this nitric oxide-mediated pathway and induces the expression of heat shock proteins to improve folding. This will be addressed in future studies.

Finally, given the fact that peroxisomal disorders are neurological diseases it is interesting to note that arginine can cross the blood–brain-barrier [34].

## Abbreviations

D3: Deuterium-labeled; D3-C22:0: Deuterium-labeled free 22,22,22-D3-docosanoic acid; DMEM: Dulbecco’s Modified Eagle’s Medium; IRD: Infantile refsum disease; MELAS: Mitochondrial encephalomyopathy lactic acidosis and stroke-like episodes; NALD: Neonatal adrenoleukodystrophy; PBD: Peroxisome biogenesis disorder; VLCFA: Very long chain fatty acids; ZSD: Zellweger spectrum disorder; ZS: Zellweger syndrome.

## Competing interests

The authors declare that they have no competing interests.

## Authors’ contributions

KB, MSE, LIJ: Conception and design, data acquisition, analysis, interpretation, manuscript draft and revision. BTPT, RJAW: conception and design, manuscript draft and revision. HRW: data interpretation, conception and design, manuscript draft and revision. All authors read and approved the final manuscript.

## Supplementary Material

Additional file 1: Figure S1 Catalase immunofluorescence of fibroblasts incubated with arginine. Two additional PEX1-G843D patient fibroblasts were incubated for 7, 14 and 21 days with arginine, glycerol and glutamine. Glycerol was used as a positive control. In four independent experiments, two cover slips per condition were examined for the number of cells with catalase-containing peroxisomes among at least 200 cells. Data are presented as means ± SD, statistical analysis was performed with a two-tailed Student’s *t*-test, *, P < 0.05; **, P < 0.005; ***, P < 0.0005; ****, P < 0.0001 versus untreated PEX1-G843D fibroblasts. Control showed 100% peroxisome positive cells. PEX1-I700fsX41 (negative control) showed no peroxisome positive cells. Click here for file
