# Supplementary figures and images for: Arginine improves peroxisome functioning in cells from patients with a mild peroxisome biogenesis disorder
Source: Orphanet J Rare Dis. 2013 Sep 9;8:138. doi: 10.1186/1750-1172-8-138 (PMC3844471; doi:10.1186/1750-1172-8-138)

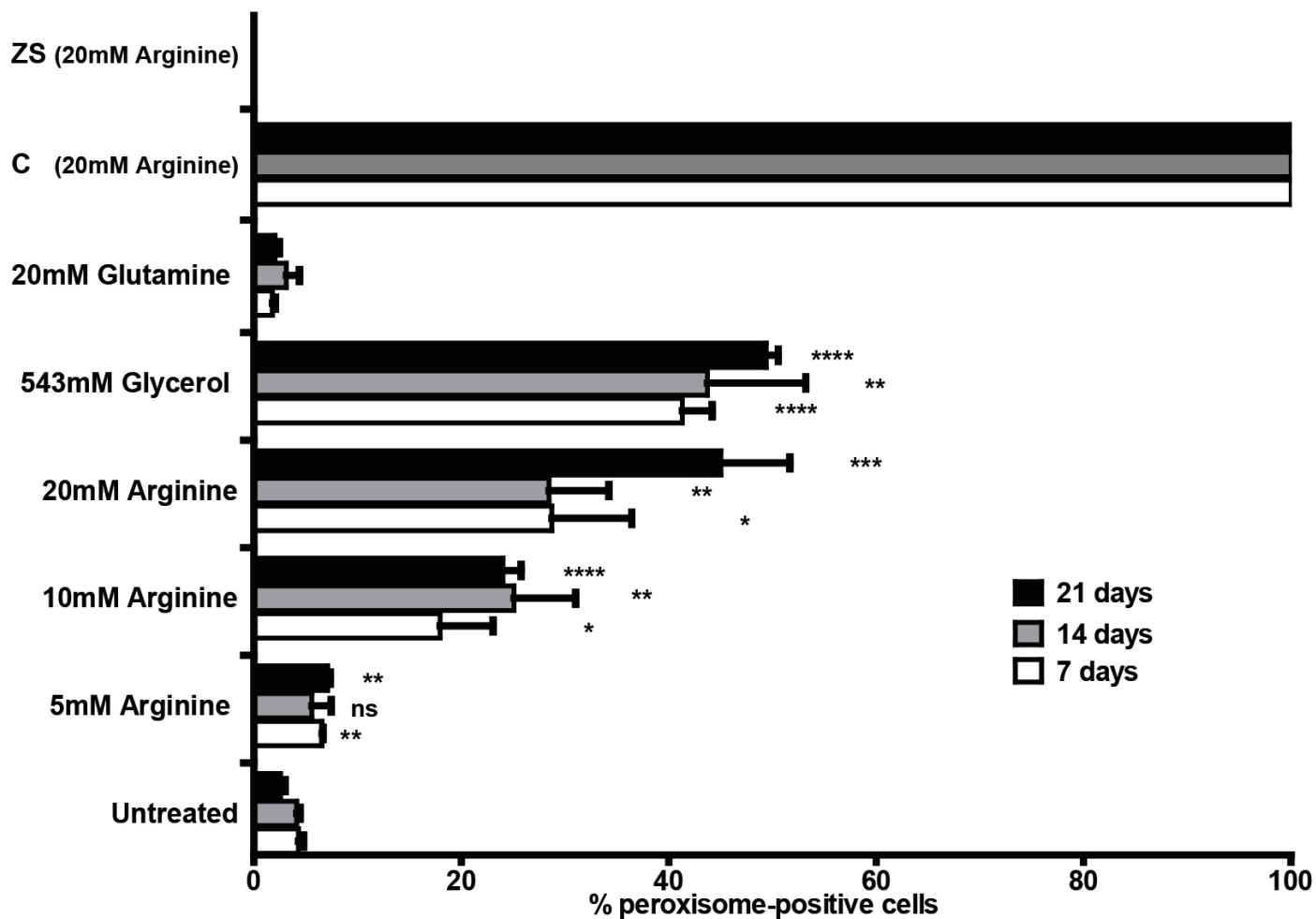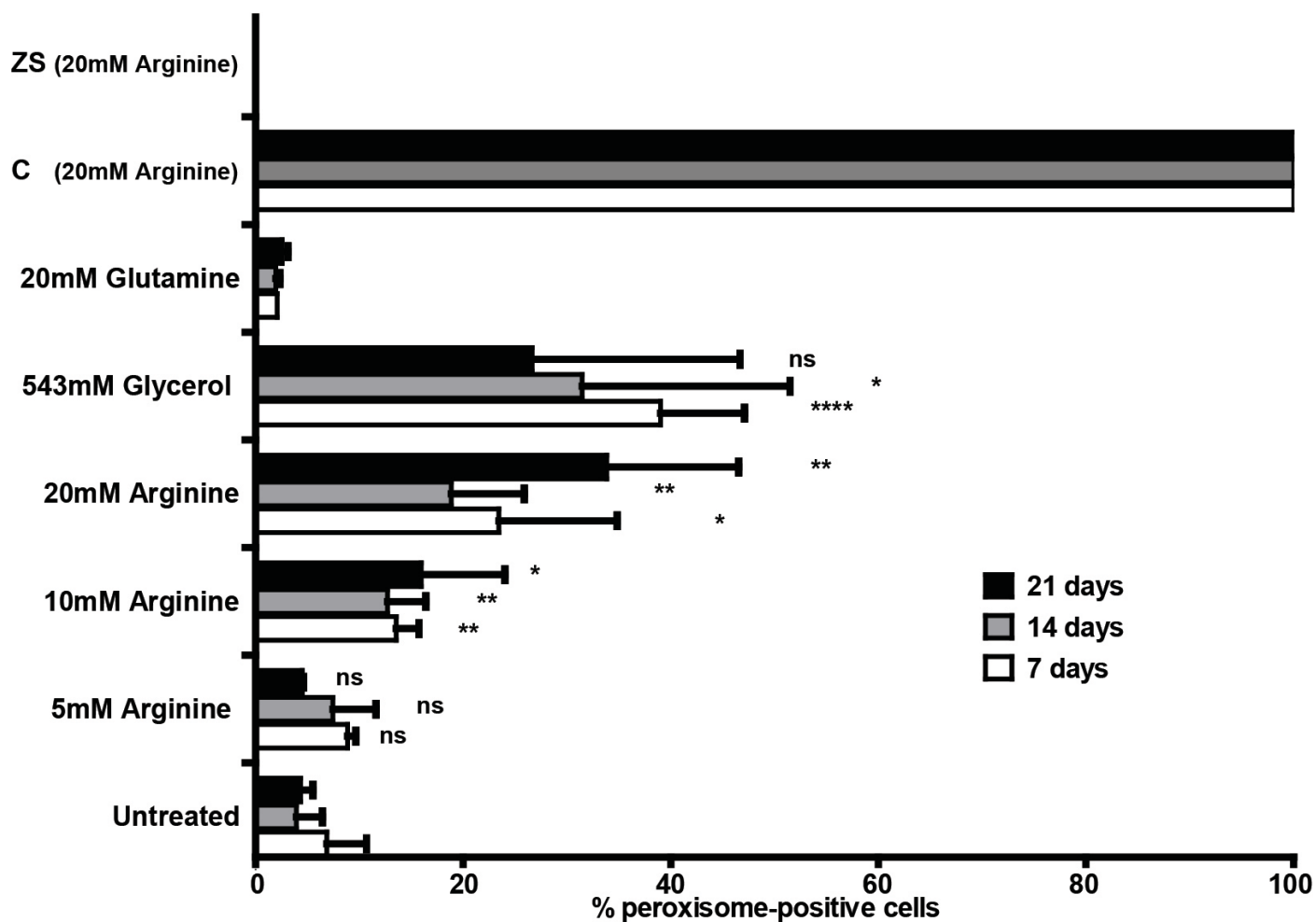

Supplement: Additional file 1: Figure S1 — Catalase immunofluorescence of fibroblasts incubated with arginine. Two additional PEX1-G843D patient fibroblasts were incubated for 7, 14 and 21 days with arginine, glycerol and glutamine. Glycerol was used as a positive control. In four independent experiments, two cover slips per condition were examined for the number of cells with catalase-containing peroxisomes among at least 200 cells. Data are presented as means ± SD, statistical analysis was performed with a two-tailed Student’s t-test, *, P < 0.05; **, P < 0.005; ***, P < 0.0005; ****, P < 0.0001 versus untreated PEX1-G843D fibroblasts. Control showed 100% peroxisome positive cells. PEX1-I700fsX41 (negative control) showed no peroxisome positive cells. [file 1750-1172-8-138-S1.pdf]
